# Revolutionizing Relief: Cervical Radiculopathy With Neurological Deficits Rescued by Cervical Disc Replacement

**DOI:** 10.7759/cureus.59923

**Published:** 2024-05-08

**Authors:** Rahul Singh, Sohael Khan, Ratnakar E Ambade, Kashyap Kanani, Vipul Agrawal, Siddharth K Patel

**Affiliations:** 1 Department of Orthopaedics, Jawaharlal Nehru Medical College, Datta Meghe Institute of Higher Education and Research, Wardha, IND; 2 Department of Orthopedics, Jawaharlal Nehru Medical College, Datta Meghe Institute of Higher Education and Research, Wardha, IND

**Keywords:** spinal surgery, surgical intervention, cervical disc replacement, neurological deficits, cervical radiculopathy

## Abstract

Cervical radiculopathy is a common condition characterized by neck pain radiating to the upper and lower limbs, often accompanied by tingling sensations, numbness, and weakness. We present the case of a 32-year-old male who presented with left-sided cervical radiculopathy and neurological deficits. Clinical examination revealed left C5/C6/C7 hypoesthesia, diminished grip strength, reduced power in the left upper and lower extremities, and a positive Spurling test. Magnetic resonance imaging (MRI) of the cervical spine revealed multilevel cervical disc herniations at C4-C5 and C5-C6 levels, resulting in stenosis. The patient underwent anterior cervical discectomies with artificial disc replacement (cervical disc arthroplasty (CDA)) at the C5-C6 level. The surgical procedure was uneventful, and the patient experienced prompt relief from neurological symptoms within two weeks postoperatively. Follow-up radiographs at one week post-surgery demonstrated a preserved range of motion at each operated level with the artificial disc in situ. This case highlights the successful management of cervical radiculopathy with neurological deficits using anterior cervical discectomy and artificial disc replacement. The timely intervention led to the resolution of symptoms and restoration of function, demonstrating the efficacy of this surgical approach in alleviating radicular symptoms and preserving cervical spine mobility. Further studies and long-term follow-up are warranted to validate the long-term outcomes and durability of artificial disc replacement in such cases.

## Introduction

Cervical radiculopathy is characterized by compression or inflammation of the nerve roots emerging from the cervical spine. It can arise from various pathologies, including disc herniation, osteophyte formation, or foraminal stenosis [1]. The symptoms of cervical radiculopathy may include radiating pain, numbness, tingling, and weakness in the upper extremities, depending on the specific nerve root(s) affected [2]. The condition can significantly impact an individual's quality of life and functional abilities, necessitating appropriate management strategies. The traditional surgical approach for treating cervical radiculopathy has been anterior cervical discectomy and fusion (ACDF). This procedure involves removing the offending disc material and fusing the adjacent vertebral bodies, thereby decompressing the affected nerve root(s) [3]. While ACDF has demonstrated efficacy in relieving symptoms, it also carries potential drawbacks, such as the loss of segmental mobility, increased stress on adjacent levels, and the risk of pseudarthrosis or adjoining segment disease [4,5]. In recent years, cervical disc arthroplasty (CDA) has emerged as an alternative to ACDF for managing sub-axial (C3-C7) cervical disc disease [6,7]. CDA involves replacing the diseased disc with an artificial disc prosthesis, preserving segmental motion and potentially reducing the risk of adjacent segment degeneration [8]. Several randomized controlled trials and systematic reviews have demonstrated comparable or superior clinical outcomes with CDA compared to ACDF for appropriately selected patients [9,10]. However, the role of CDA in managing disc herniations at the C2-C3 level remains uncertain due to the upper cervical spine's unique biomechanical characteristics and anatomical constraints [11]. The C2-C3 level is subject to increased mobility and complex loading patterns, which may pose challenges for artificial disc prostheses' long-term performance and durability [12,13].

Additionally, the vertebral artery's proximity and the foramen's narrow dimensions at this level can complicate the surgical approach and increase the risk of complications [14]. Despite these concerns, some studies have reported successful outcomes with CDA for treating C2-C3 disc herniations, particularly in carefully selected cases [15,16]. The decision to pursue CDA at this level often involves carefully weighing potential benefits, such as preserving motion and reducing adjacent segment degeneration, against the possible risks and technical challenges associated with the procedure [17,18]. In this context, we present a case report of cervical disc herniations involving the C5-C6 level, which was treated with CDA. This case highlights the potential application of CDA in managing disc herniations at the C5-C6 level. It contributes to the ongoing discussion regarding this surgical approach's appropriate indications and limitations.

## Case presentation

A 32-year-old male presented with a three-week history of neck pain radiating to the left upper and lower limbs, associated with tingling sensations and numbness. His symptoms had gradually worsened despite conservative management for over six months. Examination revealed left C5/C6/C7 hypoesthesia, reduced power in the left upper limb (extensors 3+, flexors 4 on the Medical Research Council (MRC) scale), and diminished left finger grip strength (50% compared to the right). Left lower limb power was graded 4/5.

**Figure 1:**
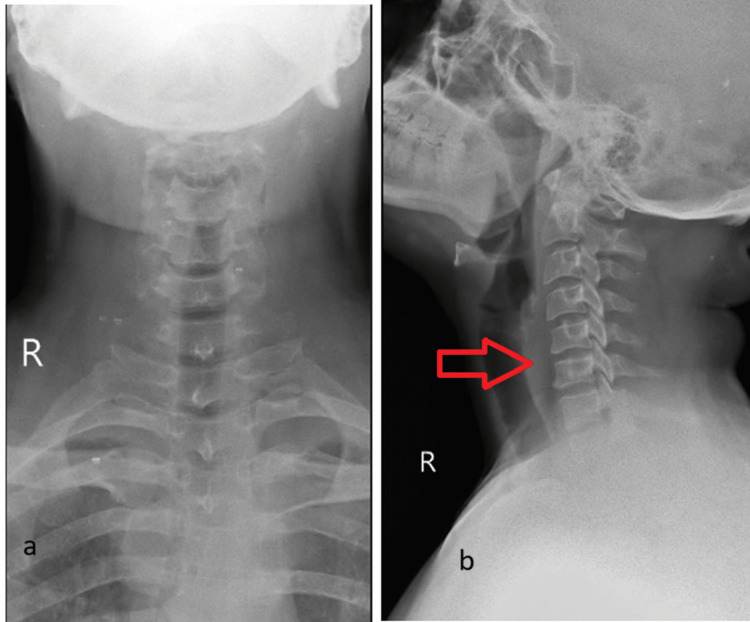
Radiographs of the cervical spine ((a) anteroposterior and (b) lateral views) showing bridging syndesmophytes, loss of cervical lordosis, and reduced space between C5 and C6 (arrow)

Radiographs of the cervical spine show reduced space between the C5 and C6 vertebrae with sclerosis of the end plate of the vertebrae as shown in Figure 1.

**Figure 2:**
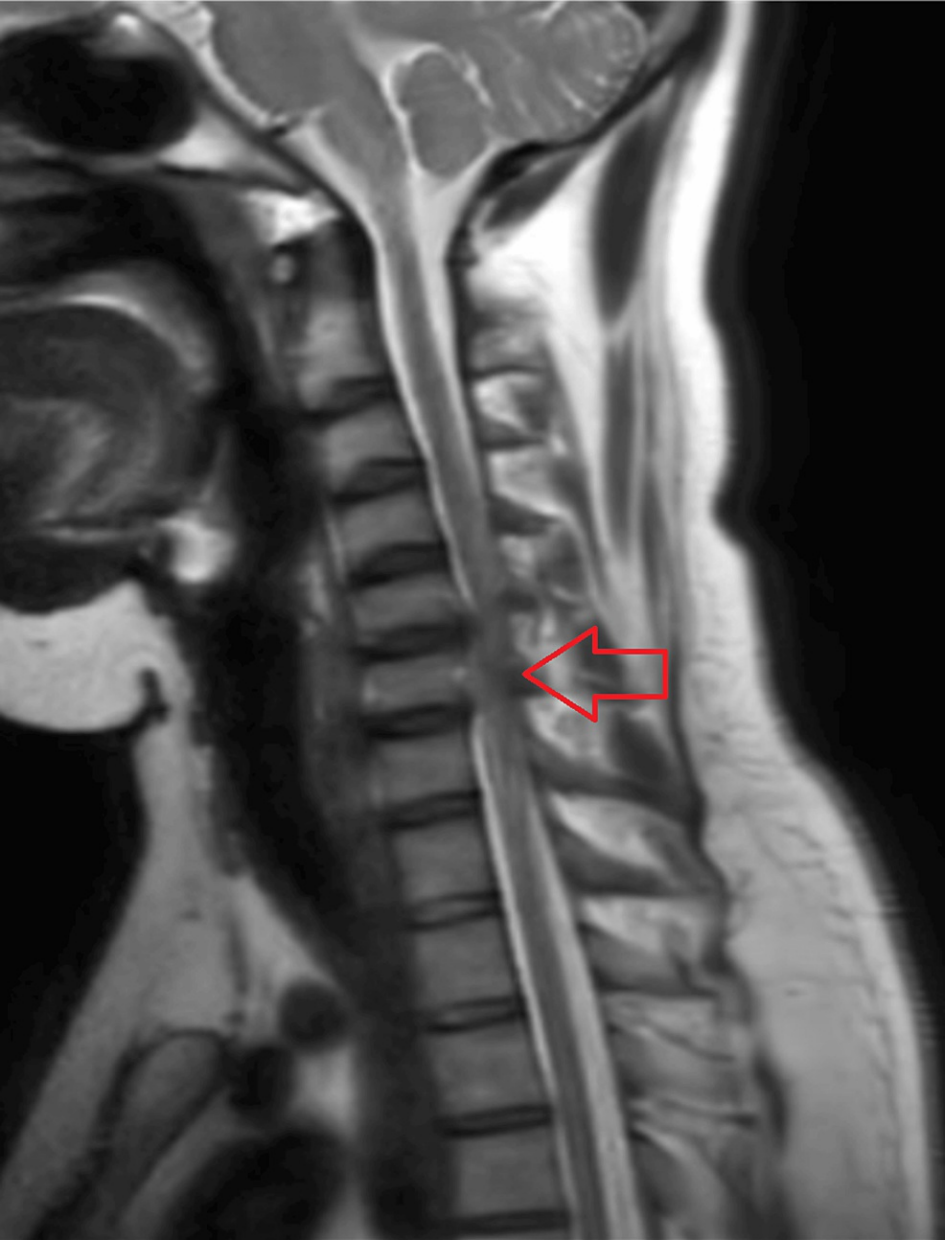
MRI of the cervical spine showing herniated discs at C4-C5 and C5-C6 causing stenosis (arrow) MRI: magnetic resonance imaging

**Figure 3:**
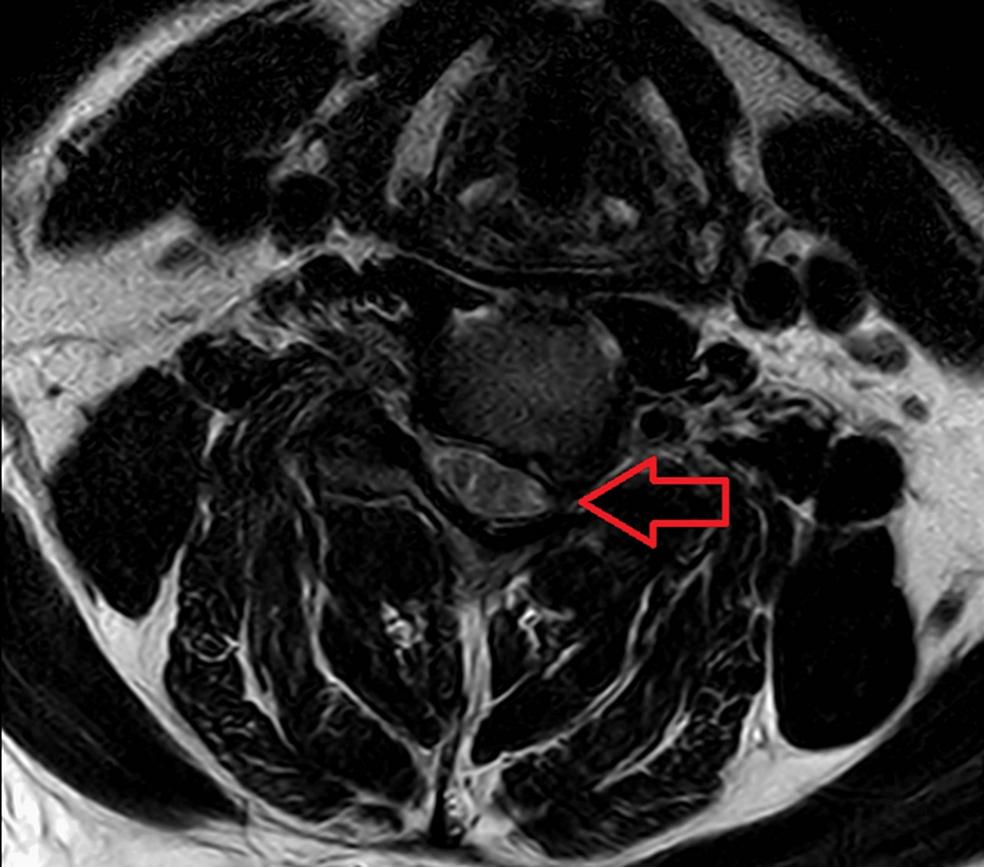
MRI (axial section) at the level of C5-C6 showing narrowing of the neural foramen causing compression of exiting and traversing nerve roots (arrow) MRI: magnetic resonance imaging

Magnetic resonance imaging (MRI) shows a disc bulge at C5-C6, indenting the thecal sac and encroaching the neural foramen as shown in Figure 2 and Figure 3.

On the right side, a horizontal incision was made in the anterior part of the neck. The muscles and tissues were carefully retracted to expose the C4-C5 and C5-C6. Discectomy at each affected level (C4-C5 and C5-C6) removed a portion of the intervertebral disc, including the herniated disc material. The surrounding bone and ligaments are carefully released to create space for the artificial disc implant.

Endplate preparation

The cartilaginous endplates of the vertebral bodies at each level are prepared to make a smooth surface for the artificial disc implant. A filer was used to remove a thin layer of bone to prepare the endplates.

Artificial disc implantation

A size 7 artificial disc implant was selected based on preoperative measurements and the patient's anatomy. The artificial disc is carefully inserted into the disc space at the C5-C6 level, replacing the removed disc material. The proper positioning and alignment of the artificial disc implants were checked using intraoperative X-rays.

**Figure 4:**
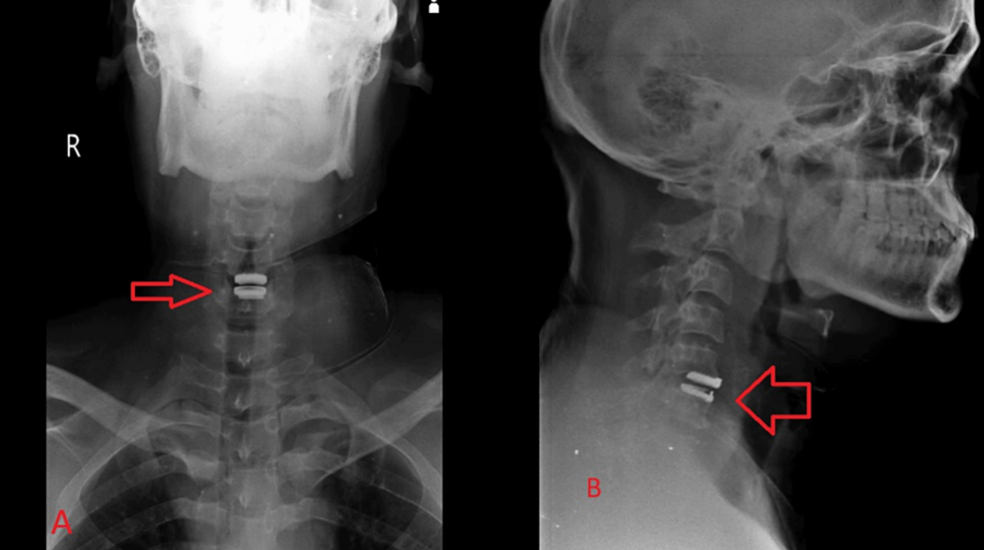
Radiographs ((A) anteroposterior and (B) lateral views) on immediate postoperative day 2 showing artificial disc at the level of C5-C6 with good alignment and placement (arrows)

Physical therapy and rehabilitation were done gradually in the form of neck range of movement exercises, and the patient had significant improvement in pain and cervical range of movement. An immediate postoperative radiograph was done and shows artificial disc placement at the C5-C6 level with good alignment and placement as shown in Figure 4.

**Figure 5:**
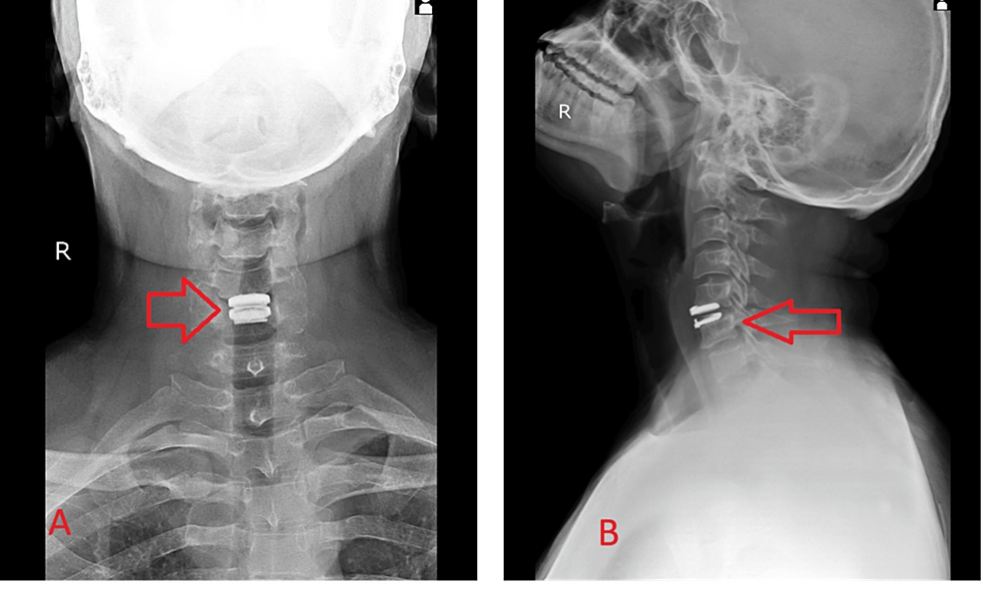
Radiographs done at three months of follow-up ((A) anteroposterior and (B) lateral views) showing a well-placed disc at the C5-C6 level with good alignment (arrows)

The patient came for follow-up after three months and had a significant reduction in radiculopathy with complete recovery of the neurological symptoms and improved neck range of movements. Radiographs were done, and the cervical disc was seen at the C5-C6 level as shown in Figure 5.

## Discussion

This case illustrates successful decompression and cervical disc arthroplasty (CDA) at the C5-C6 level in a patient with multilevel cervical disc herniations. CDA at C5-C6 has been reported very less, and its role remains to be determined due to the low incidence of disc herniation at this level and the technical challenges of the surgical approach [6,7]. However, the literature suggests segmental mobility at C5-C6 in normal individuals, with an average range of 6.1° compared to 9.7° at the more mobile C5-C6 level. While ACDF has been the gold standard for cervical disc disease [9], CDA aims to preserve segmental mobility and has shown comparable efficacy and safety to ACDF in the sub-axial cervical spine for single- and two-level disease [3-5,10]. Our patient had no contraindications to CDA such as ossification of the posterior longitudinal ligament, trauma, or significant spondylosis [11]. Preoperative imaging confirmed adequate mobility, and intraoperative findings were favorable for CDA. The main surgical challenge was achieving the optimal trajectory and exposure for C5-C6 discectomy and arthroplasty. Various anterior approaches have been described, including transoral and standard anterior cervical approaches [6,12-15]. We utilized the familiar anterior cervical approach. Placing a Caspar pin perpendicular to the C5 lower endplate facilitated accurate centering and inserting the artificial disc at C5-C6 [1]. Meticulous endplate preparation, sizing, and centering were crucial for optimal implant function [16]. Although not previously reported, our results suggest that CDA may be a viable option for C5-C6 disc herniation in appropriately selected patients without significant contraindications. The effectiveness and safety of two-level CDA have been demonstrated in Food Drug Administration trials [17,18]. Retrospective studies support its feasibility with satisfactory outcomes and maintained mobility [19,20]. In our case, the segmental range of motion was preserved at operated levels for up to six months postoperatively.

## Conclusions

This case report presents a successful cervical discectomy and artificial disc replacement at the C5-C6 level in a 32-year-old patient. Despite the patient's relatively young age, the decision to pursue cervical disc arthroplasty was made after carefully considering the patient's clinical presentation, radiographic findings, and absence of contraindications for motion-preserving surgery. The surgical technique was performed meticulously, with attention to proper endplate preparation, an appropriately sized implant selection, and accurate centering of the artificial disc. Postoperative radiographic assessments confirmed adequate decompression and preservation of segmental mobility at the treated level. This case highlights the potential applicability of cervical disc arthroplasty in younger patients who meet the appropriate clinical and radiographic criteria such as age of typically under 60-65 years old; symptoms such as neck or arm pain, weakness, and numbness; disc degeneration (radiological confirmation of disc disease); disc height and alignment (adequate for implant placement); absence of severe instability; failure of conservative treatment; and patient lifestyle (assessment of activity level and demands). While the long-term outcomes of this procedure in this age group remain to be fully elucidated, the successful early results in this case are encouraging. It is important to emphasize that cervical disc arthroplasty is not a panacea and should be approached with caution, particularly in younger patients. Careful patient selection, adherence to established indications and contraindications, and meticulous surgical technique are paramount to achieving favorable outcomes. Factors such as cervical deformities, instability, or ossification of the posterior longitudinal ligament may preclude cervical disc arthroplasty in favor of traditional fusion procedures.

Additionally, the long-term durability of artificial disc implants and the potential for adjacent segment degeneration or implant-related complications remain areas of ongoing investigation. More extensive prospective studies with extended follow-up periods are needed to fully assess cervical disc arthroplasty's long-term efficacy and safety, particularly in younger patient populations. In summary, this case report demonstrates the successful implementation of cervical discectomy and artificial disc replacement at the C5-C6 level in a 32-year-old patient. While the early results are promising, continued vigilance and long-term follow-up are necessary to better understand this approach's potential benefits and limitations in younger patient populations. Moreover, adherence to established guidelines and meticulous surgical technique remain paramount in achieving optimal outcomes with this procedure.
